# Supplementary material for: Rapid Response of Nitrogen Cycling Gene Transcription to Labile Carbon Amendments in a Soil Microbial Community
Source: mSystems. 2021 May 11;6(3):e00161-21. doi: 10.1128/mSystems.00161-21 (PMC8125072; doi:10.1128/mSystems.00161-21)
Supplement: TABLE S1 [file mSystems.00161-21-st001.pdf]

Process	Gene Name	Product Name [E.C.]
a) Assimilatory nitr	*NIT-6*	nitrite reductase (NAD(P)H) [EC:1.7.1.4]
	nirA	ferredoxin-nitrite reductase [EC:1.7.7.1]
	nasB	assimilatory nitrate reductase electron transfer subunit [EC:1.7.99.-]
	narB	ferredoxin-nitrate reductase [EC:1.7.7.2]
	nasA	assimilatory nitrate reductase catalytic subunit [EC:1.7.99.-]
	NR	nitrate reductase (NAD(P)H) [EC:1.7.1.1 1.7.1.2 1.7.1.3]
b) Complete nitrific	*narG, narZ, nxrA*	nitrate reductase / nitrite oxidoreductase, alpha subunit [EC:1.7.5.1 1.7.99.-]
	narH, narY, nxrB	nitrate reductase / nitrite oxidoreductase, beta subunit [EC:1.7.5.1 1.7.99.-]
	hao	hydroxylamine dehydrogenase [EC:1.7.2.6]
	pmoA-amoA	methane/ammonia monooxygenase subunit A [EC:1.14.18.3 1.14.99.39]
	pmoB-amoB	methane/ammonia monooxygenase subunit B
	pmoC-amoC	methane/ammonia monooxygenase subunit C
c) Denitrification	*nirK*	nitrite reductase (NO-forming) [EC:1.7.2.1]
	narG, narZ, nxrA	nitrate reductase / nitrite oxidoreductase, alpha subunit [EC:1.7.5.1 1.7.99.-]
	narH, narY, nxrB	nitrate reductase / nitrite oxidoreductase, beta subunit [EC:1.7.5.1 1.7.99.-]
	narI, narV	nitrate reductase gamma subunit [EC:1.7.5.1 1.7.99.-]
	nosZ	nitrous-oxide reductase [EC:1.7.2.4]
	norC	nitric oxide reductase subunit C
	napA	periplasmic nitrate reductase NapA [EC:1.7.99.-]
	napB	cytochrome c-type protein NapB
	norB	nitric oxide reductase subunit B [EC:1.7.2.5]
	nirS	nitrite reductase (NO-forming) / hydroxylamine reductase [EC:1.7.2.1 1.7.99.1]
	CYP55	fungal nitric oxide reductase [EC:1.7.1.14]
d) Dissimilatory nit	*nirB*	nitrite reductase (NADH) large subunit [EC:1.7.1.15]
	nirD	nitrite reductase (NADH) small subunit [EC:1.7.1.15]
	narG, narZ, nxrA	nitrate reductase / nitrite oxidoreductase, alpha subunit [EC:1.7.5.1 1.7.99.-]
	narH, narY, nxrB	nitrate reductase / nitrite oxidoreductase, beta subunit [EC:1.7.5.1 1.7.99.-]
	narI, narV	nitrate reductase gamma subunit [EC:1.7.5.1 1.7.99.-]
	napA	periplasmic nitrate reductase NapA [EC:1.7.99.-]
	napB	cytochrome c-type protein NapB
	nrfA	nitrite reductase (cytochrome c-552) [EC:1.7.2.2]
	nrfH	cytochrome c nitrite reductase small subunit
e) Glutamate Dehy	*gudB, rocG*	glutamate dehydrogenase[EC:1.4.1.2]
	GLUD1_2, gdhA	glutamate dehydrogenase (NAD(P)+) [EC:1.4.1.3]
	gdhA	glutamate dehydrogenase (NADP+) [EC:1.4.1.4]
	GDH2	glutamate dehydrogenase [EC:1.4.1.2]
f) GS-GOGAT	*GLT1*	glutamate synthase (NADH) [EC:1.4.1.14]
	gltB	glutamate synthase (NADPH) large chain [EC:1.4.1.13]

	gltD	glutamate synthase (NADPH) small chain [EC:1.4.1.13]
	GLU, gltS	glutamate synthase (ferredoxin) [EC:1.4.7.1]
	glnA, GLUL	glutamine synthetase [EC:6.3.1.2]
g) Nitrate assimilat	*NRT, narK, nrtP, nas*	MFS transporter, NNP family, nitrate/nitrite transporter
	nrtA, nasF, cynA	nitrate/nitrite transport system substrate-binding protein
	nrtB, nasE, cynB	nitrate/nitrite transport system permease protein
	nrtC, nasD	nitrate/nitrite transport system ATP-binding protein [EC:3.6.3.-]
	nrtD, cynD	nitrate/nitrite transport system ATP-binding protein
h) Nitrification	*hao*	hydroxylamine dehydrogenase [EC:1.7.2.6]
	pmoA-amoA	methane/ammonia monooxygenase subunit A [EC:1.14.18.3 1.14.99.39]
	pmoB-amoB	methane/ammonia monooxygenase subunit B
	pmoC-amoC	methane/ammonia monooxygenase subunit C
i) Nitrogen fixation	*anfG*	nitrogenase delta subunit [EC:1.18.6.1]
	nifD	nitrogenase molybdenum-iron protein alpha chain [EC:1.18.6.1]
	nifH	nitrogenase iron protein NifH
	nifK	nitrogenase molybdenum-iron protein beta chain [EC:1.18.6.1]
	vnfD	vanadium-dependent nitrogenase alpha chain [EC:1.18.6.2]
	vnfK	vanadium-dependent nitrogenase beta chain [EC:1.18.6.2]
	vnfG	vanadium nitrogenase delta subunit [EC:1.18.6.2]
	vnfH	vanadium nitrogenase iron protein
j) N Regulation	*gnlD*	[protein-PII] uridylyltransferase [EC:2.7.7.59]
	glnB	nitrogen regulatory protein P-II 1
	glnK	nitrogen regulatory protein P-II 2
	glnL; ntrB	nitrogen regulation sensor histidine kinase
	glnG; ntrC	nitrogen regulation response regulator
