# Supplementary figures and images for: Rapid Response of Nitrogen Cycling Gene Transcription to Labile Carbon Amendments in a Soil Microbial Community
Source: mSystems. 2021 May 11;6(3):e00161-21. doi: 10.1128/mSystems.00161-21 (PMC8125072; doi:10.1128/mSystems.00161-21)

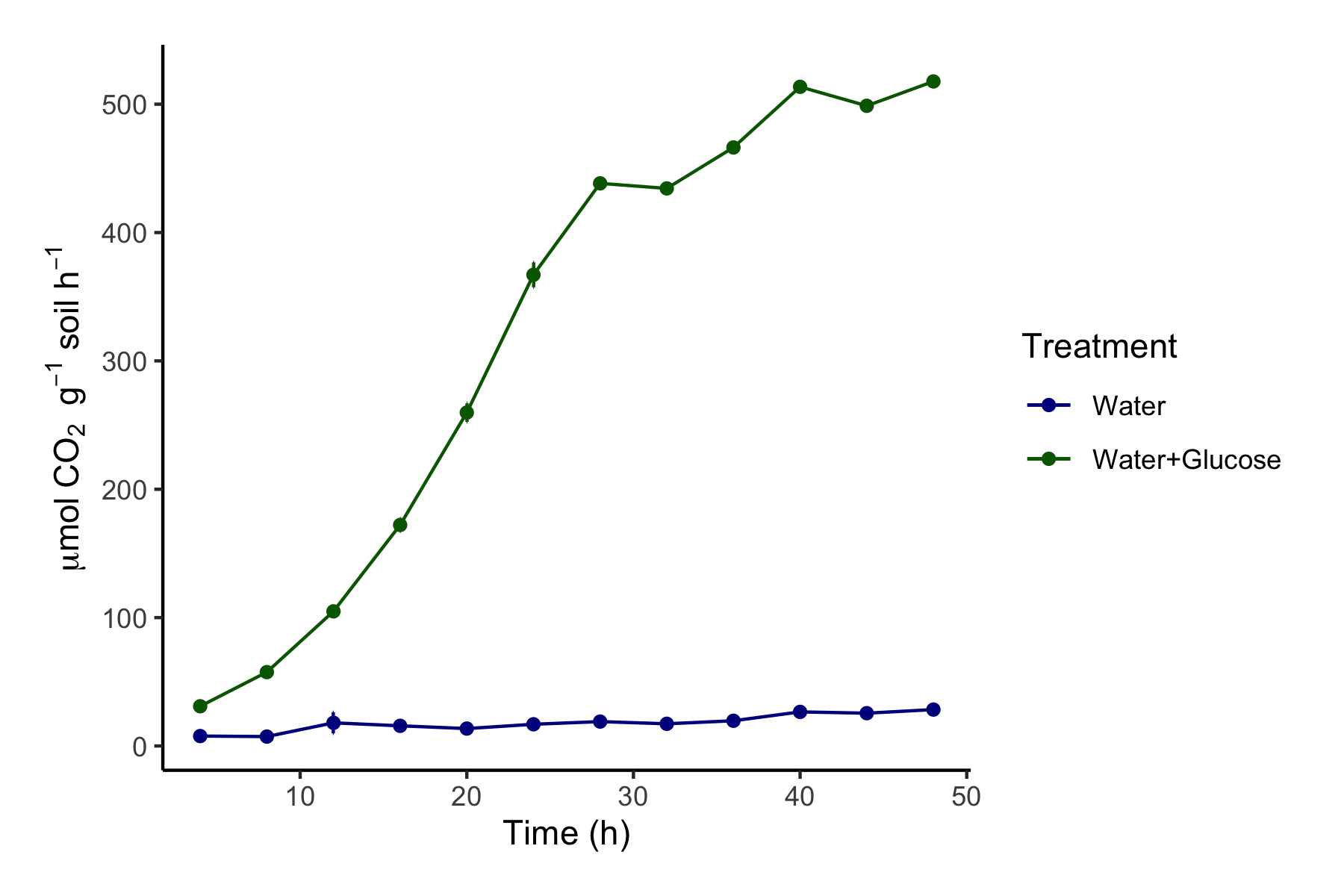

Supplement: FIG S1 [file mSystems.00161-21-sf001.tif]

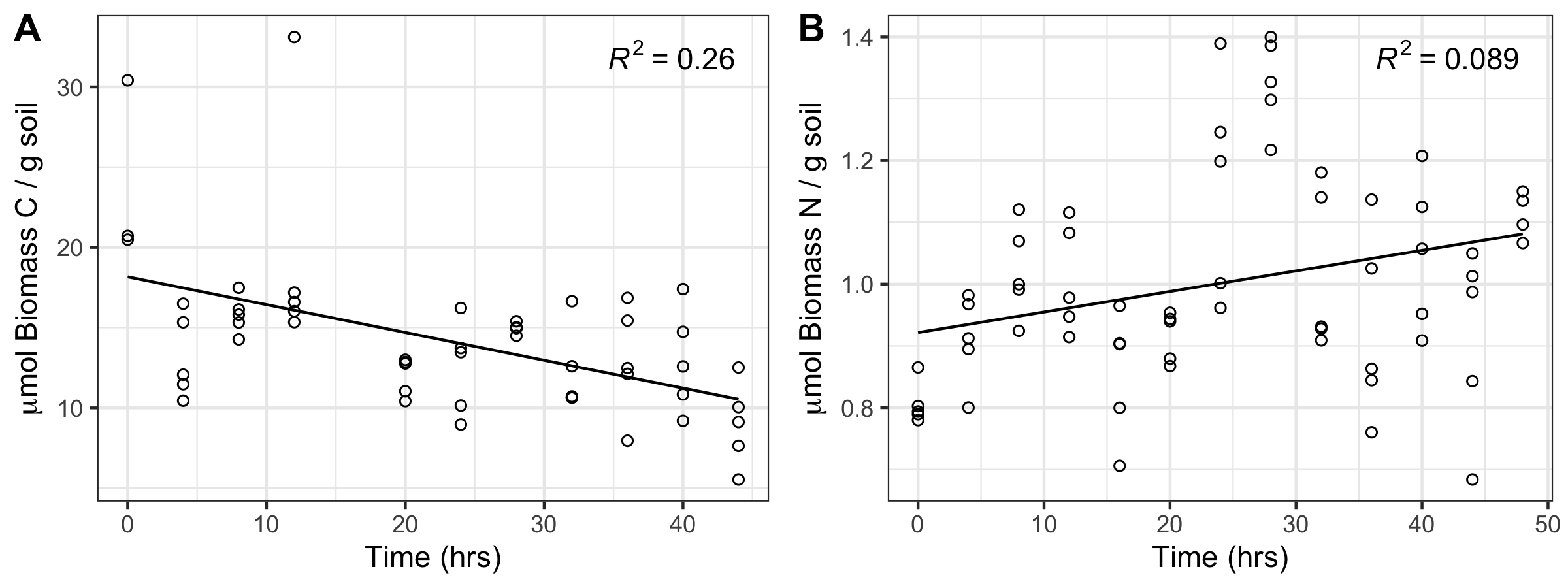

Supplement: FIG S2 [file mSystems.00161-21-sf002.tif]

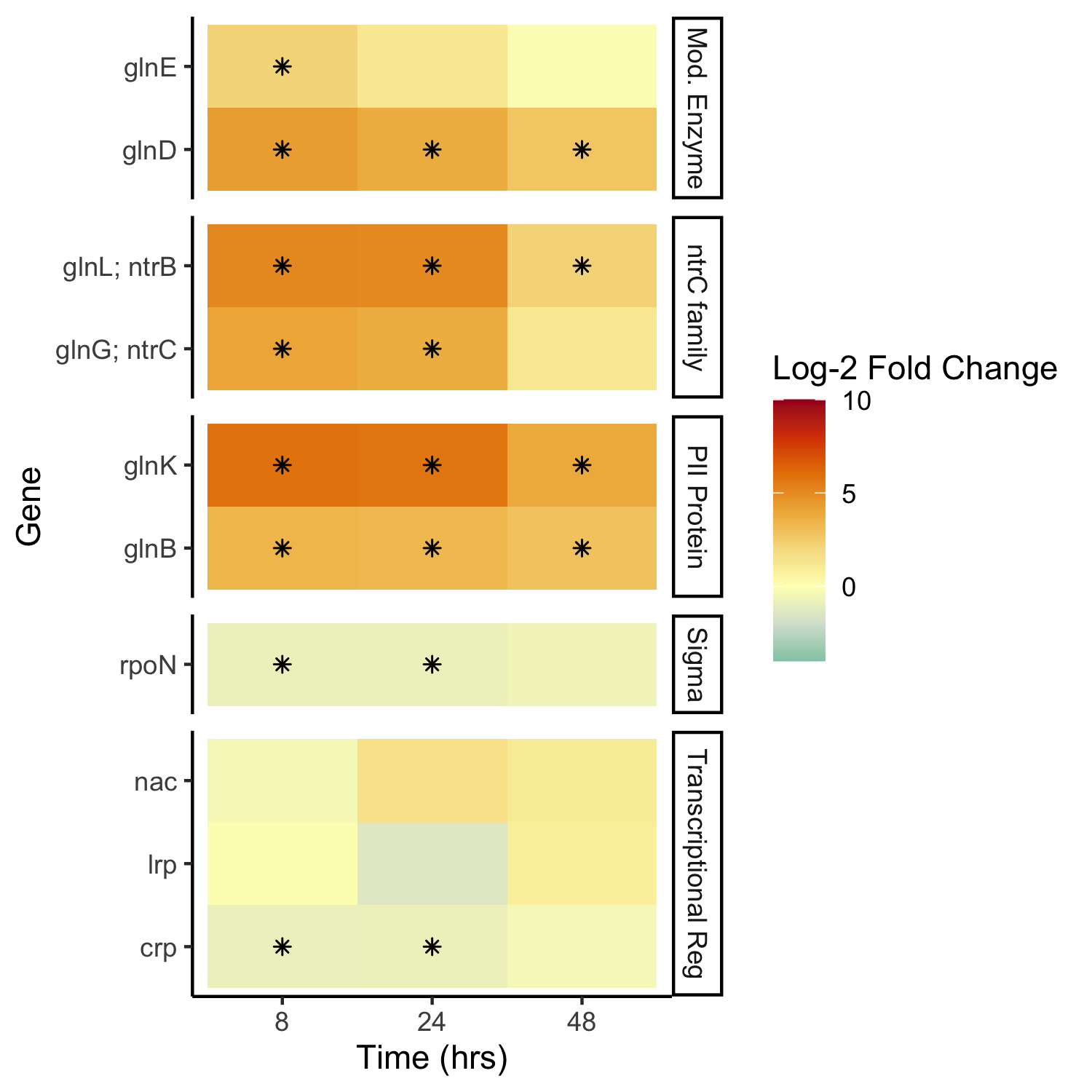

Supplement: FIG S3 [file mSystems.00161-21-sf003.tif]

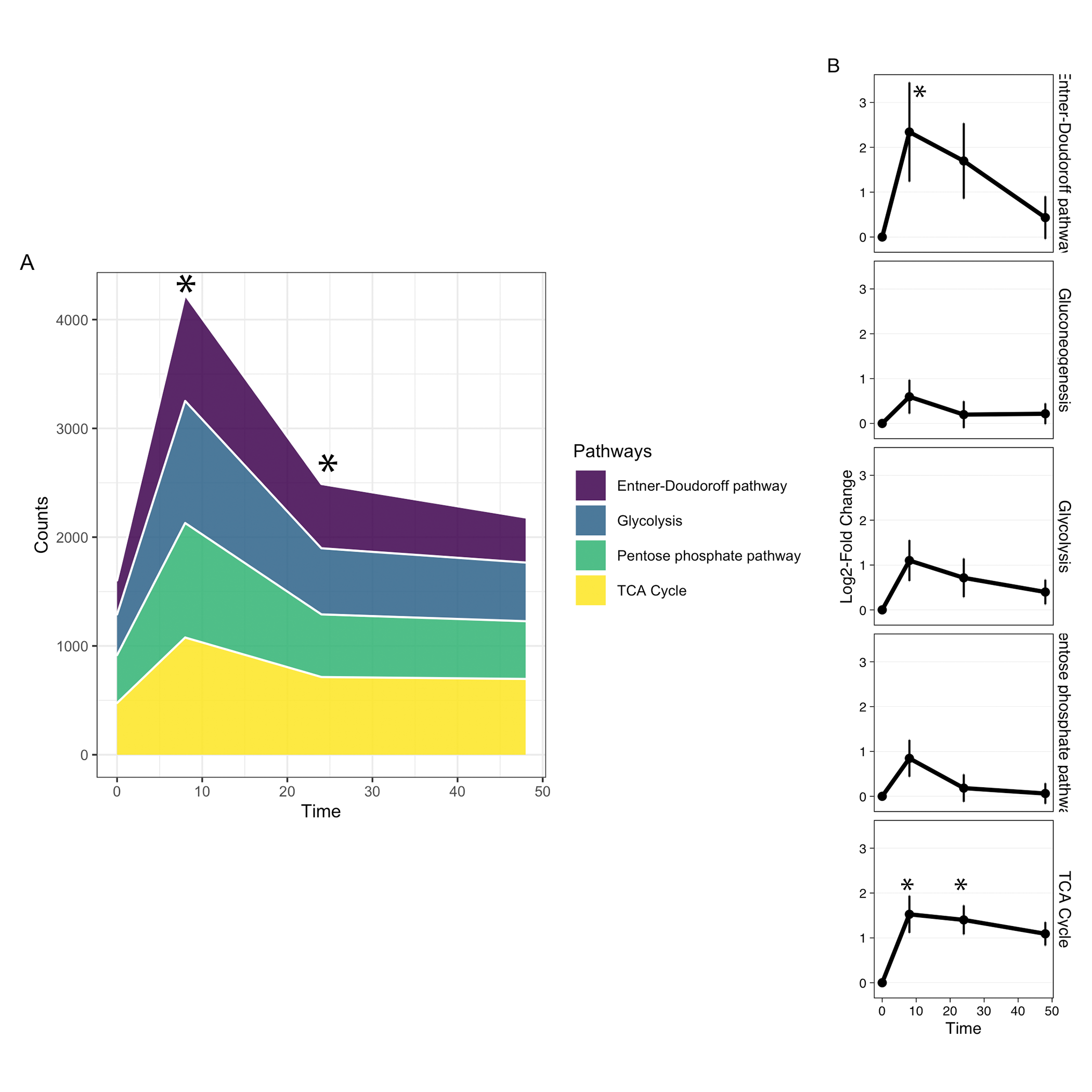

Supplement: FIG S4 [file mSystems.00161-21-sf004.tif]
