# Supplementary material for: Rapid Response of Nitrogen Cycling Gene Transcription to Labile Carbon Amendments in a Soil Microbial Community
Source: mSystems. 2021 May 11;6(3):e00161-21. doi: 10.1128/mSystems.00161-21 (PMC8125072; doi:10.1128/mSystems.00161-21)
Supplement: TABLE S3 [file mSystems.00161-21-st003.pdf]

A)

	KO Number	Gene	SIMPER % Dissimilarity
t_8 vs t_0			
	K03043	*rpoB*	0.11
	K03046	*rpoC*	0.11
	K06994	*RND superfamily*	0.1
t_{24} vs t_0			
	K02529	*lacI, galR*	0.15
	K10117	*msmE*	0.14
	K17318	*lplA*	0.14
t_{48} vs t_0			
	K02529	*lacI, galR*	0.15
	K02004	*ABC.CD.P*	0.14
	K10117	*msmE*	0.13

B)

KO_number	Gene	Description	Time (h)	Normalized Counts		se	LFC		se	FDR
K06296	*gerKB*	spore germination protein KB	24	420.45	±	16.7	8.84	±	1.1	1.32E-14
K06307	*yfkQ*	spore germination protein	24	90.42	±	20.7	8.47	±	1.3	2.60E-09
K10017	*hisP*	histidine transport system ATP-binding protein	24	66.67	±	10.9	8.04	±	1.3	2.61E-08
K03488	*licT, bglG*	beta-glucoside operon transcriptional antiterminator	24	65.76	±	16.9	8.00	±	1.3	3.10E-08
K06297	*gerKC*	spore germination protein KC	24	424.27	±	63.5	7.87	±	1.0	3.20E-13
K07717	*ycbA, glnK*	two-component system, sensor histidine kinase YcbA	24	55.77	±	2.9	7.78	±	1.3	1.50E-07
K18701	*arsC*	arsenate-mycothiol transferase	24	54.89	±	5.5	7.75	±	1.4	1.61E-06
K06311	*yndE*	spore germination protein	24	53.74	±	6.2	7.72	±	1.3	1.93E-07
K02251	*comQ*	competence protein ComQ	24	53.14	±	10.4	7.70	±	1.4	9.69E-07
K10015	*hisM*	histidine transport system permease protein	24	52.03	±	4.3	7.68	±	1.4	3.05E-06
