# Supplementary material for: Rapid Response of Nitrogen Cycling Gene Transcription to Labile Carbon Amendments in a Soil Microbial Community
Source: mSystems. 2021 May 11;6(3):e00161-21. doi: 10.1128/mSystems.00161-21 (PMC8125072; doi:10.1128/mSystems.00161-21)
Supplement: TABLE S2 [file mSystems.00161-21-st002.pdf]

Process	KO Number	Gene	Base Count	Log$_2$ Fold Change ± SE		
				Hour 8	Hour 24	Hour 48
Ammonium Transport	K03320	*amt*	26803	**4.028 ± 0.395**	**3.946 ± 0.395**	**3.259 ± 0.395**
Assimilatory nitrate reduction	K00360	*nasB*	134	9.118 ± 1.076	**8.407 ± 1.078**	**7.731 ± 1.079**
	K17877	*NIT-6*	48	**8.082 ± 1.120**	**6.589 ± 1.126**	**4.927 ± 1.138**
	K10534	*NR*	24	**6.909 ± 1.082**	**6.142 ± 1.089**	***2.909 ± 1.146***
	K00372	*nasA*	1096	**5.757 ± 0.441**	**5.484 ± 0.442**	**4.432 ± 0.442**
	K00367	*narB*	48	**7.295 ± 1.095**	**6.263 ± 1.099**	**3.542 ± 1.124**
	K00366	*nirA*	337	**4.802 ± 0.805**	**5.545 ± 0.806**	**6.420 ± 0.805**
Nitrification	K10946	*pmoC-amoC*	8186	-1.587 ± 0.897	-1.667 ± 0.897	-0.490 ± 0.897
	K10945	*pmoB-amoB*	2929	-1.331 ± 0.691	**-2.187 ± 0.692**	-0.856 ± 0.691
	K10944	*pmoA-amoA*	3928	*-2.125 ± 0.745*	**-2.365 ± 0.746**	-1.122 ± 0.745
	K10535	*hao*	111	**-2.475 ± 0.518**	**-2.687 ± 0.529**	***-1.394 ± 0.520***
	K00371	*narH, narY, nxrB*	1570	**-2.088 ± 0.533**	**-2.348 ± 0.533**	***-1.282 ± 0.533***
	K00370	*narG, narZ, nxrA*	2020	**-2.322 ± 0.597**	**-2.177 ± 0.597**	-0.492 ± 0.597
Denitrification	K15864	*nirS*	25	-0.986 ± 0.619	*1.578 ± 0.603*	**1.933 ± 0.600**
	K04561	*norB*	43	*-1.004 ± 0.505*	0.001 ± 0.509	-0.179 ± 0.511
	K02568	*napB*	12	-1.049 ± 1.160	*2.834 ± 1.126*	1.138 ± 1.145
	K02567	*napA*	176	**-0.834 ± 0.242**	**1.159 ± 0.240**	**0.927 ± 0.241**
	K02305	*norC*	17	-0.357 ± 0.618	0.916 ± 0.620	0.972 ± 0.621
	K00376	*nosZ*	85	**-1.999 ± 0.345**	-0.509 ± 0.345	-0.139 ± 0.343
	K00374	*narI, narV*	47	0.073 ± 0.512	**3.286 ± 0.495**	**3.513 ± 0.494**

	K00371	*narH, narY, nxrB*	1570	**-2.088**	±	**0.533**	**-2.348**	±	**0.533**	***-1.282***	±	***0.533***
	K00370	*narG, narZ, nxrA*	2020	**-2.322**	±	**0.597**	**-2.177**	±	**0.597**	-0.492	±	0.597
	K00368	*nirK*	3823	**-1.888**	±	**0.584**	**-2.652**	±	**0.584**	***-1.362***	±	***0.584***
DNRA	K15876	*nrfH*	10	-0.390	±	0.756	-1.253	±	0.808	0.493	±	0.767
	K03385	*nrfA*	34	*-1.049*	±	*0.421*	-0.612	±	0.431	-0.720	±	0.434
	K02568	*napB*	12	-1.049	±	1.160	*2.834*	±	*1.126*	1.138	±	1.145
	K02567	*napA*	176	**-0.834**	±	**0.242**	**1.159**	±	**0.240**	**0.927**	±	**0.241**
	K00374	*narI, narV*	47	0.073	±	0.512	**3.286**	±	**0.495**	**3.513**	±	**0.494**
	K00371	*narH, narY, nxrB*	1570	**-2.088**	±	**0.533**	**-2.348**	±	**0.533**	***-1.282***	±	***0.533***
	K00370	*narG, narZ, nxrA*	2020	**-2.322**	±	**0.597**	**-2.177**	±	**0.597**	-0.492	±	0.597
	K00363	*nirD*	873	**6.184**	±	**0.481**	**6.306**	±	**0.481**	**6.065**	±	**0.481**
	K00362	*nirB*	3230	**7.702**	±	**0.421**	**7.426**	±	**0.421**	**6.538**	±	**0.421**
GDH	K00262	*EC 1.4.1.4, gdhA*	111	**1.542**	±	**0.312**	0.494	±	0.320	-0.108	±	0.325
	K00261	*EC 1.4.1.3 GLUD1_2, gdhA*	469	**-0.991**	±	**0.299**	*-0.837*	±	*0.301*	-0.532	±	0.300
	K00260	*EC 1.4.1.2 gudB, rocG*	26	-0.809	±	0.899	*2.326*	±	*0.884*	***2.229***	±	***0.884***
	K15371	*EC 1.4.1.2 GDH2*	310	0.820	±	0.433	**1.350**	±	**0.434**	0.696	±	0.435
GS-GOGAT	K01915	*glnA, GLUL*	26238	**2.809**	±	**0.314**	**2.395**	±	**0.314**	**1.819**	±	**0.314**
	K00284	*GLU, gltS*	1376	**2.412**	±	**0.230**	**1.604**	±	**0.231**	**0.930**	±	**0.232**
	K00266	*gltD*	1491	**2.962**	±	**0.310**	**1.781**	±	**0.310**	**1.075**	±	**0.311**
	K00265	*gltB*	3365	**3.762**	±	**0.289**	**3.048**	±	**0.289**	**2.295**	±	**0.289**
	K00264	*GLT1*	68	**3.656**	±	**0.513**	**3.458**	±	**0.518**	**2.059**	±	**0.527**
Nitrate assimilation	K15579	*nrtD, cynD*	4	2.792	±	1.739	*3.859*	±	*1.747*	2.931	±	1.769
	K15578	*nrtC, nasD*	474	**9.289**	±	**1.051**	**9.549**	±	**1.051**	**7.181**	±	**1.053**
	K15577	*nrtB, nasE, cynB*	610	**8.921**	±	**0.935**	**9.277**	±	**0.935**	**7.470**	±	**0.936**
	K15576	*nrtA, nasF, cynA*	1493	**10.200**	±	**0.968**	**10.556**	±	**0.968**	**9.010**	±	**0.969**

	K02575	*NRT, narK, nrtP, nasA*	1447	**6.643**	**±**	**0.514**	**6.875**	**±**	**0.514**	**5.552**	**±**	**0.515**
Nitrogen fixation	K02591	*nifK*	25	-1.209	±	1.693	**7.529**	**±**	**1.410**	***3.941***	**±**	***1.437***
	K02588	*nifH*	203	-0.598	±	1.512	**8.980**	**±**	**1.441**	2.648	±	1.464
	K02586	*nifD*	54	1.049	±	1.622	**8.684**	**±**	**1.557**	***3.640***	**±**	***1.589***
